# The Prognostic Value of the Prognostic Nutritional Index in Patients with Advanced or Metastatic Gastric Cancer Treated with Immunotherapy

**DOI:** 10.3390/nu15194290

**Published:** 2023-10-08

**Authors:** Yuting Pan, Yue Ma, Guanghai Dai

**Affiliations:** 1 Chinese PLA Medical School, Beijing 100853, China; 301oncpyt@sina.com (Y.P.); 301oncmy@sina.com (Y.M.); 2 Medical Oncology Department, The First Medical Center, Chinese People’s Liberation Army General Hospital, Beijing 100853, China

**Keywords:** prognostic nutritional index, immunotherapy, advanced gastric cancer, prognostic factor, survival outcome

## Abstract

In recent years, the therapeutic effect of monoclonal antibodies against programmed cell death protein-1 (PD-1) in patients with locally advanced or metastatic gastric or gastroesophageal junction (G/GEJ) cancer has been confirmed in many studies. The exploration and discovery of new biomarker combinations based on tumor characteristics and tumor microenvironment help screen superior patients and realize precise immunotherapy. As an evaluation index of immunonutritional status, the prognostic nutritional index (PNI) is low cost, simple and easy to obtain, and effective in determining the prognosis of tumor patients. We selected 268 consecutive AGC patients who were treated with ICI therapy from December 2014 to May 2021. We measured their pretreatment of the PNI levels and performed univariate and multivariate Cox regression analyses of progression-free survival (PFS) or overall survival (OS) after ICI therapy. The low pretreatment PNI level of AGC patients was significantly correlated with shorter PFS (*p* < 0.001) and OS (*p* < 0.001) after ICI treatment. In univariate and multivariate analyses of the associations between PNI and OS or PFS, PNI is an independent prognostic factor for PFS (HR = 1.511; 95%CI 1.154–1.977; *p* = 0.003) and OS (HR = 1.431; 95%CI 1.049–1.951; *p* = 0.024), respectively. Notably, decreased PNI during treatment with ICIs was associated with early relapse and death. Pretreatment with PNI might help to identify AGC patients who will obtain a survival benefit from ICI therapy.

## 1. Introduction

Gastric cancer (GC) is a worldwide clinically aggressive gastrointestinal tumor. It is the fifth most common cancer and the fourth leading cause of death from cancers in the world [1]. In China, the morbidity and mortality of GC rank second and third, respectively, seriously endangering public health. Despite the decline in morbidity and mortality over the past few decades, GC is still one of the major global health challenges [2]. The median overall survival (mOS) of advanced GC (AGC) is only about 8 months [3]. So far, both chemotherapy and targeted drugs are facing a bottleneck, and the mOS can barely exceed 2 years. Globally, the 5-year overall survival (OS) rate for AGC is only 10% to 15% [4].

As one of the breakthroughs in cancer treatment, immunotherapy has become an effective approach after surgery, chemotherapy, radiotherapy, and targeted therapy [3]. This treatment involves stimulating the immune system to control tumor growth, and it specifically targets tumor cells rather than normal ones. At present, therapeutic strategies utilizing the immune system involve checkpoint inhibitors, chimeric antigen receptor T cells (CAR T cells), monoclonal antibodies, cancer vaccines, cytokines, radiation immunotherapy, and oncolytic virus therapy [5], among which immune checkpoint inhibitors (ICIs) have always been the research focus [6]. As one of the representative drugs, pembrolizumab demonstrated its potential value in antitumor therapy in the phase I Keynote-012 study, Keynote-059 cohort 1, Keynote-659 (NCT03382600), and Keynote-811 (NCT03615326), but Keynote-061 and KEYNOTE062 were not very effective [4,7,8,9,10,11]. Another drug, nivolumab, has also shown promising antitumor effects in attract-2 studies, CheckMate 649 Phase 3 trials, and Attract-4 (NCT02746796) [12,13,14].

Although immunotherapy offers hope to patients with AGC, good results can only be achieved in specific populations. Predictive biomarkers can play a crucial role in screening patients who may benefit from selected or targeted therapies [6]. However, clinical evaluation of serum markers such as carcinoembryonic antigen (CEA), carbohydrate antigen (CA19-9), and gastric cancer antigen (CA724) to immunotherapy is limited. There is no definitive biomarker that can accurately evaluate the therapeutic effect of ICIs [4]. Recent studies have indicated that self-expressed programmed death ligand-1 (PD-L1) and microsatellite steady-state (MSI) can be used to effectively assess the treatment results of immunotherapy in patients with AGC [15,16,17,18,19]. Peripheral blood inflammation composite indicators such as lung immune prognostic index (LIPI), neutrophil–lymphocyte ratio (NLR), platelet–lymphocyte ratio (PLR), and hemoglobin (Hb) level have been proven to be convenient and promising biomarkers for GC prognosis [20,21,22,23,24,25,26].

Among numerous biomarkers that have been reported recently, peripheral blood biomarkers stand out because of their convenient and quick detection methods. In addition, due to the high heterogeneity of tumor tissue in GC, the accuracy of tumor biomarkers to evaluate treatment prognosis is not ideal, which will inevitably hinder the precise diagnosis of patients and the selection of targeted therapy [27].

Prognostic nutritional index (PNI) has attracted attention as an indicator for assessing systemic nutritional status and immunocompetence and as a prognostic biomarker. It quantifies serum albumin and peripheral blood lymphocyte counts via simple calculations, bringing many conveniences to clinicians’ nutritional and immune status assessments of patients. In recent years, studies of PNI as a prognostic indicator for various types of cancer have been reported. Fumihiro Shoji et al. studied the relationship between pretreatment c-reactive protein (CRP), neutrophil–lymphocyte ratio (NLR), and PNI and the efficacy and prognosis of immunotherapy in 102 patients with non-small cell lung cancer (NSCLC) [28]. They found that progression-free survival (PFS) and overall survival (OS) in the low PNI group were significantly shorter than those in the high PNI group, and PNI was still an independent prognostic factor of PFS after multivariate analysis [28]. Therefore, they believe that the pretreatment PNI is helpful in identifying patients with AGC who may benefit from immunotherapy [28]. Based on this belief, we retrospectively studied the relationship between pretreatment PNI and the prognosis of patients with advanced gastric cancer treated with immunotherapy.

## 2. Material and Methods

### 2.1. Research Subjects

A total of 268 patients with stage GC-IV who received ICI treatment in the Advanced Oncology Department of the General Hospital of the Chinese People’s Liberation Army from December 2014 to May 2021 were selected. Inclusion criteria: (1) The patient was diagnosed with AGC via comprehensive examination, including imaging examination, serum tumor marker examination, and histopathological biopsy; (2) the patient received at least two cycles of ICI therapy; (3) the patient completed at least one radiographic efficacy evaluation based on RECIST1.1 criteria during treatment; (4) complete blood routine and blood biochemical results within one week before the first use of ICIs; (5) <80 years old; (6) expected survival ≥ 3 months; and (7) no obvious abnormalities in blood routine, coagulation function, liver and kidney function. Exclusion criteria included the following: (1) patients who could not provide imaging data before and after ICI treatment for comparison; (2) complicated with other types of malignant tumors; (3) complicated with other diseases that may affect peripheral blood albumin and neutrophils, such as hematological diseases, infections, viral hepatitis, cirrhosis, etc.; (4) clinical, pathological, and laboratory results and other relevant data are incomplete; and (5) non-tumor causes of death. The clinical features and tumor features of patients were collected using medical record inquiry, including their smoking history, smoking exposure, sex, age, tumor type, the status of HER-2 expression, the status of liver metastasis, response to line before immunotherapy, the status of pleural fluid, the status of ascites, the number of metastatic sites, lines of treatment with ICIs, ICI agent, immunotherapy scheme, and Eastern Cooperative Oncology Group performance status scores (ECOG PS). At the same time, albumin and neutrophil values from the blood routine parameters obtained 7 days before immunotherapy were collected.

### 2.2. Treatment Regimens

According to the difference in treatment regimen, the patients were divided into immune monotherapy group and combined chemotherapy group. The types and doses of ICIs used in immunotherapy are as follows: (1) the recommended intravenous infusion of sintilimab is 200 mg once every 3 weeks; (2) the recommended intravenous infusion of riprilizumab is 3 mg/kg once every 2 weeks; (3) the recommended intravenous infusion of Pembrolizumab is 2 mg/kg over 30 min at one time once for every 3 weeks; and (4) the recommended intravenous injection of nivolumab is 3 mg/kg or at the fixed dose of 240 mg once every 2 weeks. The first imaging assessment of nivolumab was performed 2–4 weeks after the 3rd intravenous injection, while the assessments of toripalimab, sintilimab, and pembrolizumab were conducted 3–5 weeks after the 2nd intravenous injection. The 3-week dosing regimen for trastuzumab is initial loading dose of 8 mg/kg, followed by 6 mg/kg once every 3 weeks. The infusion time was about 90 min when the 6 mg/kg dose was repeated once every three weeks. If the patient showed good tolerance at the initial infusion, the subsequent infusion might be changed to 30 min. The recommended dose of Ipilimumab is 1 mg/kg once every 6 weeks for 30 min intravenous infusion 30 min intravenous infusion of combined 360 mg nivolumab once every 3 weeks, or 3 mg/kg nivolumab once every 2 weeks. Chemotherapy regimen included: (1) XELOX regimen: 130 mg/m^2^ oxaliplatin on the first day; from day 1 to day 14, capecitabine (850–1250 mg/m^2^) was taken twice a day after breakfast and dinner, and the regimen was repeated every 3 weeks. (2) SOX regimen: intravenous drip of 130 mg/m^2^ oxaliplatin on the first day; tiggio capsule (40–60 mg) was taken twice a day after breakfast and dinner for 2 weeks, and then suspended for 7 days, forming a 21 days treatment course; (3) DCF regimen: intravenous injection of 75 mg/m^2^ docetaxel on the first day of each course, which should be finished in 1 h; continuous intravenous drip of 75 mg/m^2^ cisplatin on the first day of each course; continuous intravenous drip of 750 mg/m^2^ fluorouracil on the 1st to 5th day. One treatment course was repeated every 21 days. (4) PF regimen: intravenous drip of 25 mg/m^2^ cisplatin on the 1st to 3rd day; continuous intravenous infusion of 500 mg/m^2^ 5-fluorouracil on the 1st to 5th day, one chemotherapy cycle every 4 weeks; (5) FOLFOX regimen: intravenous infusion of 85 mg/m^2^ oxaliplatin for 2 h on the first day; intravenous infusion of 200 mg/m^2^ leucovorin for 2 h on the 1st and 2nd days; intravenous injection of 400 mg/m^2^ fluorouracil on the 1st and 2nd days, together with the drip of 600 mg/m^2^ fluorouracil for 22 h. The treatment was suspended for 1 week after 14 days; (6) Combination of irinotecan and oxaliplatin: intravenous injection of irinotecan (180 mg/m^2^) and oxaliplatin (130 mg/m^2^) on the first day of each 14-day session. (7) Combination of irinotecan and raltitrexed: intravenous injection of irinotecan (180 mg/m^2^) and raltitrexed (3 mg/m^2^) on the first day of each 14-day session. (8) Others: Each patient was assigned a treatment plan according to the case stage and general health status.

### 2.3. Assessment

RECIST1.1 criteria were adopted to assess the efficacy, including complete response (CR), partial response (PR), stable disease (SD), and progressive disease (PD). The short-term efficacy was evaluated by disease control rate (DCR) = (CR + PR + SD)/total cases × 100%, and overall response rate (ORR) = (CR+PR)/total cases × 100%. For long-term efficacy evaluation, progression-free survival (PFS) was defined as the time from the first treatment to the confirmation of PD, death, or the last follow-up, and OS was defined as the time from the start of immunotherapy to death.

### 2.4. Pretreatment Calculation of the PNI and the PNI Cut-Off Value

PNI was calculated using the following formula: 1 × serum albumin level (g/L) + 5 × absolute lymphocyte count (10^9^/L) in the peripheral blood within one week before the first use of ICIs [29]. The serum albumin level can be queried in the biochemical test program, and its unit is g/L. The absolute lymphocyte count can be queried in the routine blood test program, and its unit is 10^9^/L. A receiver operating characteristic (ROC) curve is applied to elucidate the optimal cutoff value for PNI. We determined to take the 43.44 as the best cut-off value of PNI.

### 2.5. Statistical Analysis

All data were processed using SPSS26.0 and GraphPad Prism9. The data were summarized as the min–max range and median for non-normally distributed continuous variables. The data were reported as percentages and counts of categorical variables. Plotting the ROC curve of the immunotherapy effect at month 12 versus the pre-treatment PNI data resulted in an optimal cut-off value for PNI of 43.44. With ɑ = 0.005 and β = 0.2 is the standard to predict the survival period of two groups of patients with low PNI and high PNI. The analysis shows that the OS of low- and high-PNI groups is 8.1 months and 16.1 months, respectively. Kaplan–Meier method was used to describe the survival curve. To evaluate the value of PNI to OS and PFS, Cox regression model was used to analyze the influencing factors. Values lower than 0.05 (*p* < 0.05) were considered statistically significant.

## 3. Results

### 3.1. Baseline Characteristics

**Figure 1:**
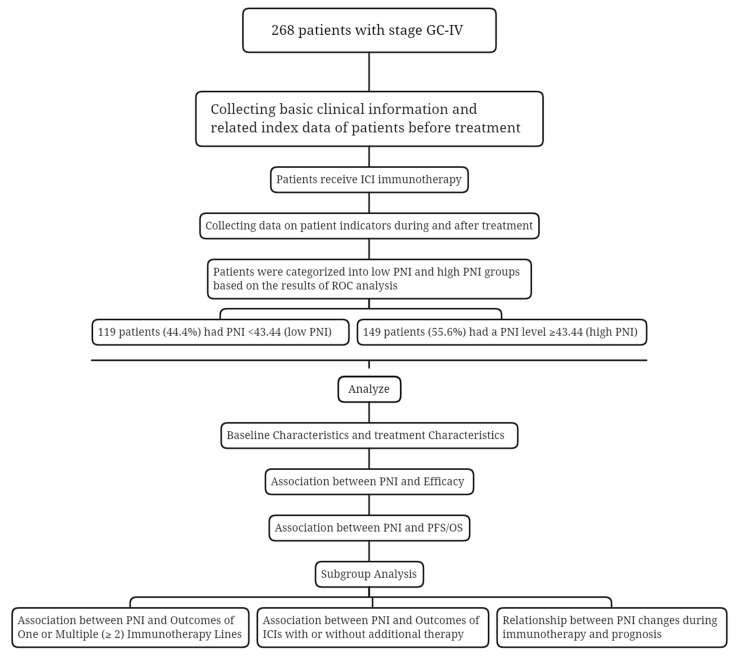
Study design flowchart. Abbreviations: GC-IV: gastric cancer IV stage; ICI: immune checkpoint inhibitors; PNI: prognostic nutritional index; ROC: receiver operating characteristic; PFS: progression-free survival; OS: overall survival.

A total of 268 AGC patients who have received ICIs participated in this study. An overview of the study for all patients is shown in Figure 1. The patients were clinically characterized by the following features shown in Table 1. Among the patients, 199 (74.3%) are male, and 69 (25.7%) are female. More than half had no history of smoking (62.3%); most had short history of smoking exposure (82.1% less than or equal to 30 years); the ECOG PS was 0-1 (94%); the tumors were located at body/fundus (41%); and there was no hepatic metastasis (55.6%), no negative expression of HER-2 (66%), no pleural fluid (92.9%), and no ascites (75.4%). After grouping according to the best cut-off value of PNI, one hundred and forty-nine (55.6%) had PNI levels ≥ 43.44 (high-PNI), and the remaining one hundred and nineteen (44.4%) patients had PNI < 43.44 (low-PNI).

### 3.2. Treatment Characteristics

In total, 125 of the 268 patients (46.6%) had first-line ICIs, and 143 (53.4%) had multi-line ICIs. Additionally, 43 patients (16%) had a combined regimen of immunization and chemotherapy, 43 patients (16%) with a combined regimen of immunization and target therapy, 130 patients (48.5%) with a combined regimen of immunization, target therapy, and chemotherapy, and 52 patients (19.4%) with the immunization without additional therapy (Table 1).

### 3.3. ROC Analysis and Grouping

**Figure 2:**
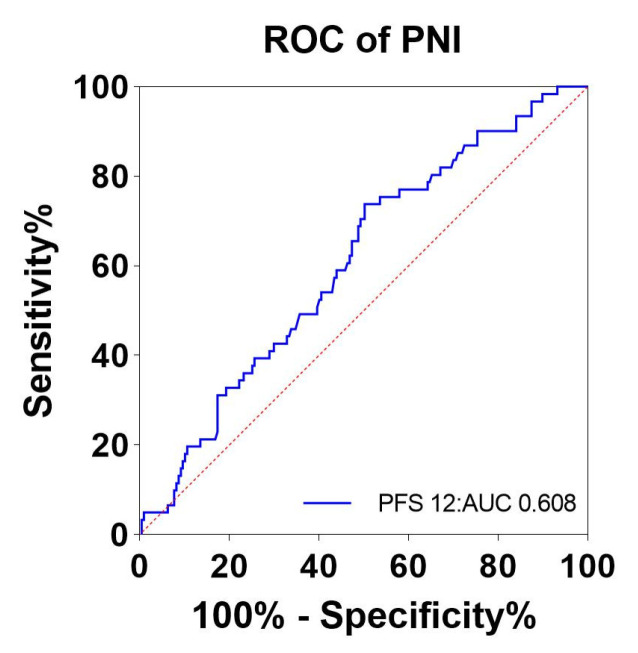
ROC curves for pretreatment PNI to assess 12-month tumor progression rates. Abbreviations: ROC: receiver operator characteristic; PNI: the prognostic nutritional index; blue line: PNI; red line: reference line.

ROC curves were plotted for the effect of immunotherapy and the level of pretreatment PNI using pretreatment PNI as the test variable and the rate of tumor progression at month 12 as the state variable. The area under the ROC curve was 0.608, indicating that the difference was statistically significant (*p* = 0.011). The optimal cutoff value for PNI was 43.44, and its corresponding sensitivity and specificity were 73.8% and 49.8%, respectively. (Figure 2). In this study population, 149 patients (55.6%) had a PNI level ≥43.44 (high PNI), and the remaining 119 patients (44.4%) had a PNI < 43.44 (low PNI).

### 3.4. Association between PNI and Efficacy

**Figure 3:**
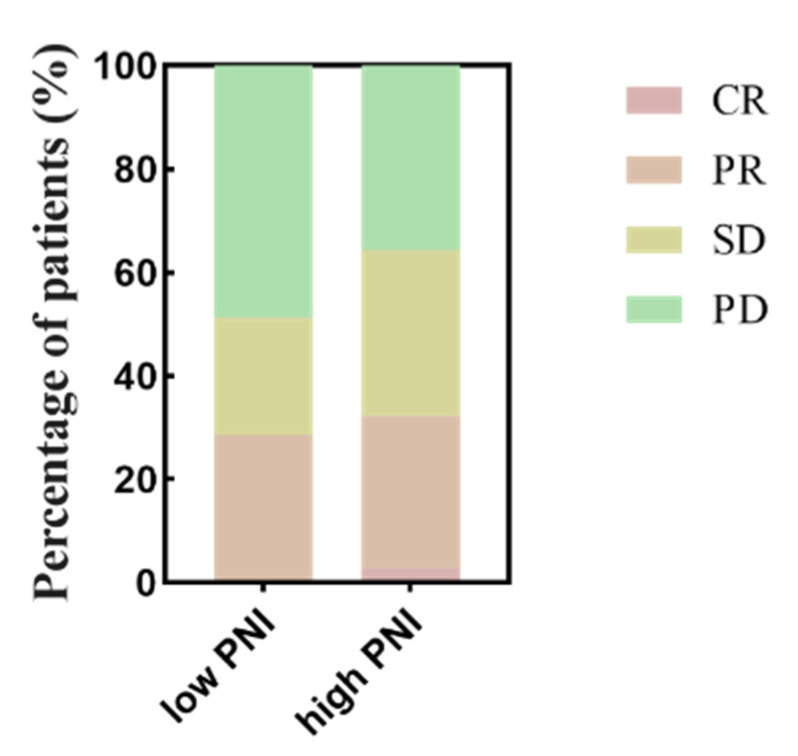
Relationship between PNI groups and response to anti-PD-1 treatment. Using the optimal cutoff value of 43.44 for PNI as a criterion, all patients were divided into two groups, low PNI and high PNI, and the percentage of different responses to anti-PD-1 treatment was calculated for each group separately. Although PD patients made up the largest percentage of patients in both groups, the percentage of PD patients was significantly higher in the low PNI group than in the high PNI group. Abbreviations: PNI: the prognostic nutritional index; PD-1: programmed cell death-1; CR: complete response; PR: partial response; SD: stable disease; PD: progressive disease.

This study evaluated the optimal efficacy of all AGC patients, the results of which were as follows: PD accounted for 41.4% (111 patients), CR 1.5% (4 patients), PR 29.1% (78 patients), and SD 28% (75 patients) (Table 2 and Figure 3). The ORR was 30.6%, and the DCR was 58.6% (Table 2). The results of the logistic regression of DCR are summarized in Table 3. There was a significant difference in DCR between the low- and high-PNI groups (51.3% vs. 64.4%, *p* = 0.034). The multivariate logistic results showed that there was a statistically significant effect of when to use immunotherapy on disease progression (OR = 0.387, 95%CI 0.231–0.648, *p* < 0.001) and that the use of immunotherapy in the second and later lines would increase the risk of disease progression. The low- and high-PNI groups did not show a clear difference in ORR (28.6% vs. 32.2%; *p* = 0.594) (Table 2).

### 3.5. Association between PNI and Clinic-Pathological Features

**Figure 4:**
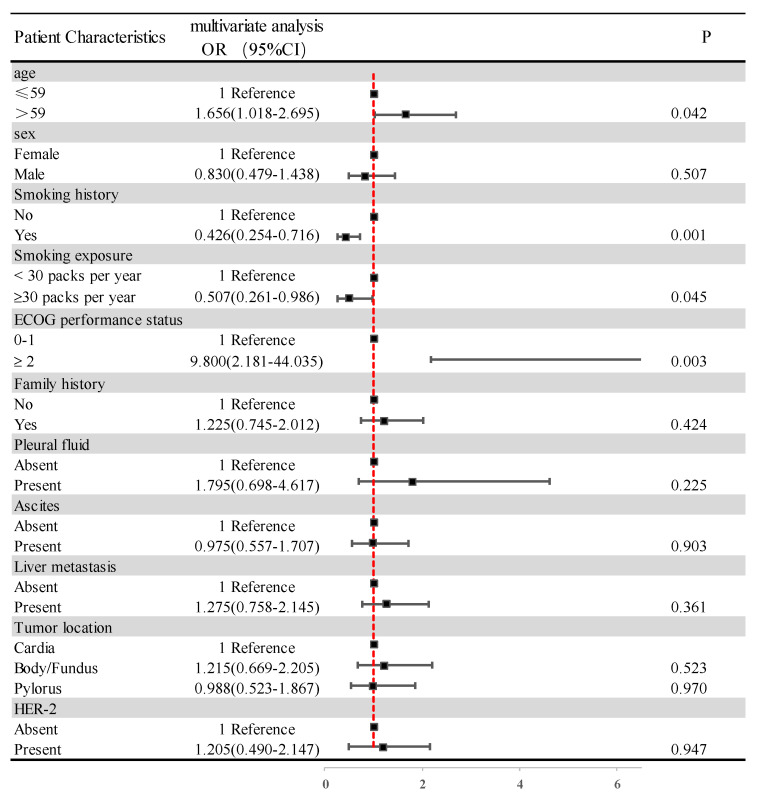
Clinical–pathological features of the patients according to the PNI. Abbreviations: ECOG PS: Eastern Cooperative Oncology Group performance status score; HER-2: human epidermal growth factor receptor-2.

We used logistic regression equations to analyze the correlation of basic characteristics, such as age, sex, smoking history, smoking exposure, ECOG PS, family history, pleural fluid, ascites, liver metastases, tumor location, HER-2 expression, and PNI, and the results are shown in Figure 4. Increasing age increases the risk of low PNI with statistical significance (OR 1.656; 95%CI 1.018–2.695; *p* = 0.042). A history of smoking reduced the risk of low PNI in a statistically significant way (OR 0.426; 95%CI 0.254–0.716; *p* = 0.001). In further analysis, smoking for more than 30 years was, likewise, statistically significant in reducing the risk of low PNI (OR 0.507 95%CI 0.261–0.986; *p* = 0.045). However, there was no correlation between other patient clinical characteristics, such as sex and PNI.

### 3.6. Association between PNI and PFS

**Figure 5:**
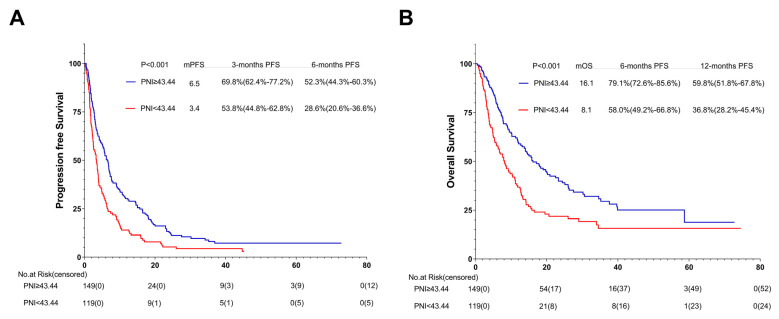
PFS (**A**) and OS (**B**) of the PNI of patients with AGC receiving ICI cohort. Survival curves of patients with AGC receiving ICI cohort were plotted using PFS and OS as outcome variables, disease progression status and survival status as status variables, and PNI as a grouping variable, respectively. Abbreviations: PFS: progression-free survival; OS: overall survival; PNI: the prognostic nutritional index; AGC: advanced gastric cancer.

**Figure 6:**
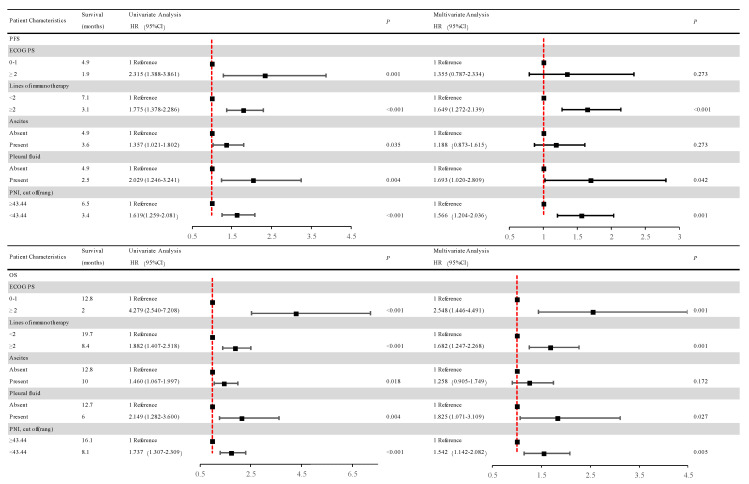
Univariate and multivariate analyses of factors associated with overall survival and progression-free survival. After COX univariate and multivariate regression analyses, patients in the low-PNI group had a high risk of disease progression and a high risk of death in the high-PNI group. Abbreviations: PFS: progression-free survival; OS: overall survival; PNI: the prognostic nutritional index; PD: progressive disease; ECOG PS: Eastern Cooperative Oncology Group performance status scores.

Regarding the last follow-up on 1 July 2021, 251 of the 268 AGC patients (93.7%) showed tumor progression. Compared with the patients in the low-PNI group, those in the high-PNI group showed a strong association with longer PFS (6.5 months vs. 3.4 months; *p* < 0.001) (Figure 5). Figure 6 summarizes the results of univariate and multivariate analyses related to PFS. As for the univariate analysis, PFS improvement was detected in the patients with good PS (ECOG PS 0-1) and no ascites or pleural effusion. For first-line ICI treatments, the results also showed improvements in PFS.

According to the multivariate analysis, patients in the low-PNI group were independently associated with an increased risk of disease progression (HR = 1.566; 95% CI, 1.204–2.036; *p* = 0.001) over 1.5-fold higher than in the high-PNI group.

Apart from the above, patients receiving ICIs after first-line treatment were independently associated with a risk of disease progression (HR = 1.649; 95% CI 1.272–2.139; *p* < 0.001) more than 1.6 times higher than the first-line ICIs group. Patients with pleural fluid were independently associated with a risk of disease progression (HR = 1.693; 95% CI 1.020–2.809; *p* = 0.042) 1.6 times higher than patients without pleural fluid.

### 3.7. Association between PNI and OS

At the last follow-up on 1 July 2021, 192 of 268 AGC patients (71.6%) passed away. Compared with patients in the low-PNI group, those in the high-PNI one were closely associated with longer OS (16.1 months vs. 8.1 months; *p* < 0.001) (Figure 5B).

Univariate and multivariate analyses relevant to OS are shown in Figure 6. The results of univariate analysis of OS are similar to those of PFS, indicating that improvement in OS was detected in patients with good PS (ECOG PS 0-1) and no ascites or pleural effusion. And first-line ICI treatment also showed improvements in OS.

Multivariate analysis revealed that the low-PNI group was independently associated with more than 1.5 times the risk of death (HR = 1.542; 95% CI 1.142–2.082; *p* = 0.005) than the high-PNI group.

In addition, patients with no pleural effusion and good PS (ECOG PS 0-1) were independently associated with OS improvement. Patients receiving first-line ICIs were also independently associated with OS improvement. Patients with pleural fluid were independently associated with a risk of death (HR = 1.825; 95% CI 1.071–3.109; *p* = 0.027) over 1.8 times higher than those without pleural fluid. Patients with good PS (ECOG PS 0-1) were independently associated with a risk of death (HR = 2.548; 95% CI 1.446–4.491; *p* = 0.001) 2.5 times higher than those with poor PS (ECOG PS ≥ 2). Patients receiving ICIs after first-line treatment were independently associated with a risk of death (HR = 1.682; 95% CI 1.247–2.268; *p* = 0.001) 1.6 times higher than the first-line ICI group.

### 3.8. Association between PNI and Outcomes of One or Multiple (≥2) Immunotherapy Lines: Subgroup Analysis

**Figure 7:**
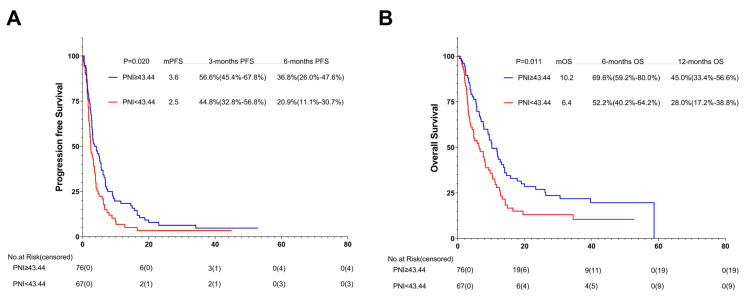
PFS (**A**) and OS (**B**) of the multi-line of patients with AGC, receiving PD-1 inhibitor cohort. Survival curves of the multi-line of patients with AGC receiving PD-1 inhibitor cohort were plotted using PFS and OS as outcome variables, disease progression status and survival status as status variables, and PNI as a grouping variable, respectively. Abbreviations: PFS: progression-free survival; OS: overall survival; PD-1: programmed cell death-1; PNI: the prognostic nutritional index.

According to the results of the multivariate analysis, the patients treated with first-line ICIs were independently associated with improved OS and PFS. The subgroup analysis was conducted based on different immunotherapy lines. Among the 143 patients with subsequent ICIs, 76 (53.1%) were in the high-PNI group and 67 (46.9%) in the low-PNI group. Both PFS and OS were improved in the high-PNI group compared with the high- and low-PNI groups (3.6 months vs. 2.5 months, *p* = 0.020; 10.2 months vs. 6.4 months, *p* = 0.011) (Figure 7A,B).

**Figure 8:**
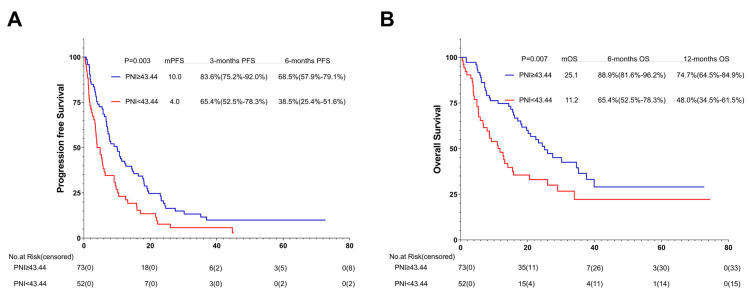
PFS (**A**) and OS (**B**) of the first-line of patients with AGC, receiving PD-1 inhibitor cohort. Survival curves of the 1st line of patients with AGC, receiving PD-1 inhibitor cohort were plotted using PFS and OS as outcome variables, disease progression status and survival status as status variables, and PNI as a grouping variable, respectively. Abbreviations: PFS: progression-free survival; OS: overall survival; PD-1: programmed cell death-1.

Among the 125 patients receiving the first-line ICI treatment, 73 (58.4%) were in the high-PNI group and 52 (41.6%) in the low-PNI group. In comparison with the PFS and OS of the high- and low-PNI groups, that of the high-PNI group improved (10.0 months vs. 4.0 months, *p* = 0.003; 25.1 months vs. 11.2 months, *p* = 0.007) (Figure 8A,B).

### 3.9. Association between PNI and Outcomes of ICIs with or without Additional Therapy: Subgroup Analysis

**Figure 9:**
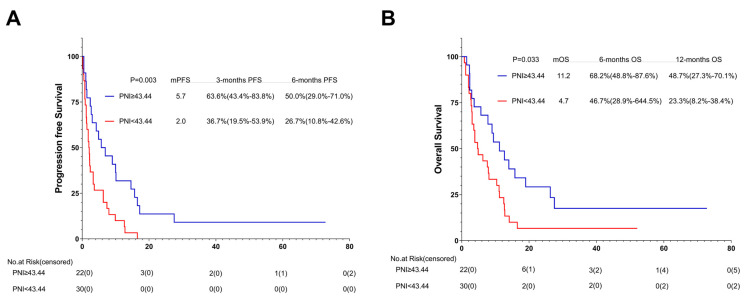
PFS (**A**) and (**B**) OS of AGC patients treated with PD-1 inhibitor combined without additional therapy. Survival curves of AGC patients treated with PD-1 inhibitor combined without additional therapy were plotted using PFS and OS as outcome variables, disease progression status and survival status as status variables, and PNI as a grouping variable, respectively. Abbreviations: PFS: progression-free survival; OS: overall survival; PD-1: programmed cell death-1.

In order to reduce the sampling error caused by using different immunotherapy schemes in different treatment lines, we also performed a subgroup analysis on the basis of different immunotherapy regimens. Among the 52 patients who received ICIs without additional therapy, 22 (42.3%) were in the high-PNI group and 30 (57.7%) in the low-PNI group. In comparison with the PFS and OS of the high- and low-PNI groups, that of the high-PNI group improved (5.7 months vs. 2.0 months, *p* = 0.003; 11.2 months vs. 4.7 months, *p* = 0.033) (Figure 9A,B).

**Figure 10:**
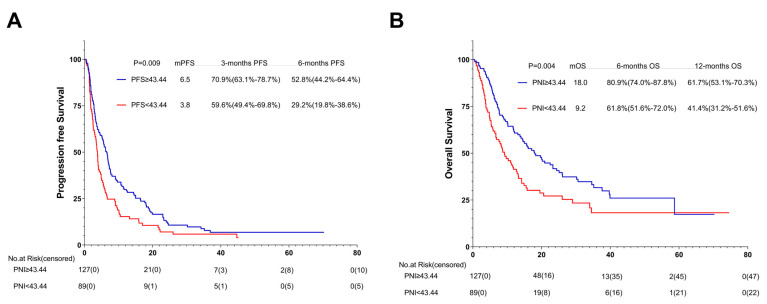
PFS (**A**) and (**B**) OS of AGC patients treated with PD-1 inhibitor combined with additional therapy. Survival curves of AGC patients treated with PD-1 inhibitor combined with additional therapy were plotted using PFS and OS as outcome variables, disease progression status and survival status as status variables, and PNI as a grouping variable, respectively. Abbreviations: PFS: progression-free survival; OS: overall survival; PD-1: programmed cell death-1.

Among the 216 patients treated with the ICIs combined with additional therapy, 127 (58.8%) were in the high-PNI group and 89 (41.2%) in the low-PNI group. In comparison with the PFS and OS of the high- and low-PNI groups, that of the high-PNI group improved (6.5 months vs. 3.8 months, *p* = 0.009; 18.0 months vs. 9.2 months, *p* = 0.004) (Figure 10A,B).

### 3.10. Relationship between PNI Changes during Immunotherapy and Prognosis: Subgroup Analysis

**Figure 11:**
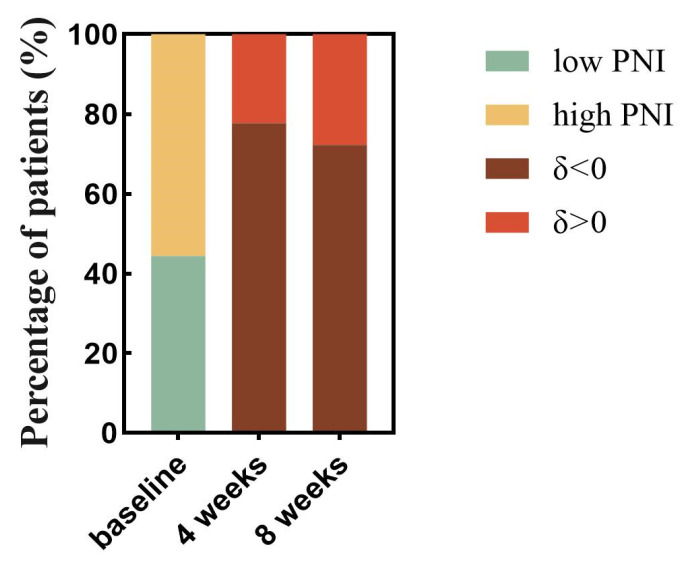
Proportional analysis of trends in PNI with time since treatment with PD-1 inhibitors. The proportion of patients in the low and high PNI groups was calculated based on baseline data of PNI before PD-1 inhibitor treatment (the lime green color represents the percentage of patients in the low PNI group, and the earthy yellow color represents the percentage of patients in the high PNI group). The proportion of patients with decreased PNI and increased PNI was then calculated based on the positive and negative grouping of δ change values derived from PNI data after 4 weeks of treatment with PD-1 inhibitors-baseline data of pretreatment PNI (tan color represents reduced PNI levels after treatment and the dark red color represents increased PNI levels after treatment). 8-week data were obtained in the same way. Abbreviations: PD-1: programmed cell death-1; δ changes: (4 weeks or 8 weeks—baseline).

After evaluating the PNI changes at different times after patients received immunotherapy in relation to disease progression, respectively, we found that after 4 weeks of immunotherapy, PNI data were available for 220 patients, of which 49 (22.3%) had δ changes (4 weeks—baseline) > 0 and 171 (77.7%) had δ changes (4 weeks—baseline) < 0. After 8 weeks of receiving immunotherapy, there were 205 patients for whom PNI data were available, of whom 57 (27.8%) had δ changes (8 weeks—baseline) > 0 and 148 (72.2%) had δ changes (8 weeks—baseline) < 0 (Figure 11). We found a statistically significant increase in the risk of recurrent tumor progression within 3 months with a decrease in the PNI index from baseline after 4 weeks of receiving immunotherapy (OR = 0.954, 95%CI 0.911–0.999, *p* = 0.043) (Table 4). A decrease in the PNI index from baseline after 8 weeks of immunotherapy was associated with a statistically significant (OR = 0.943, 95%CI 0.898–0.989, *p* = 0.017) increase in the risk of death occurring within 8 months (Table 5).

## 4. Discussion

Immunotherapy is emerging as a promising anticancer strategy for many tumors [30]. Immunotherapy for GC has achieved fruitful results and changed treatment procedures [31], showing great potential to improve the prognosis of patients [32]. Recent breakthrough studies on ICIs represented by programmed death-1 (PD-1) and PD-L1 have opened new avenues for immunotherapy of GC [17,33]. However, although anti-PD-1 antibody is a promising approach for AGC patients, its efficiency is still limited [17]. Furthermore, immunotherapy drugs are expensive and easy to develop drug resistance and hyper-progressive disease, which limits their wide application in clinical practice [34,35,36].

In order to optimize the comprehensive anti-cancer regimen of immunotherapy, it is urgent to study how biomarkers accurately identify the population with immunotherapy advantages and thus achieve as much precision and predictability as possible [37]. However, existing prediction biomarkers are still insufficient, and prediction methods need to be improved [36]. Peripheral blood inflammation composite indicators such as LIPI, NLR, PLR, and Hb levels have proved to be convenient and promising biomarkers for GC prognosis [20,21,22,23,24,25,26]. Considering the high heterogeneity of tumor tissue in GC, the accuracy of tumor biomarkers to evaluate treatment prognosis is not high, which will inevitably hinder the precise diagnosis of patients and the selection of targeted therapy [27]. In contrast, the accuracy of the composite index, which combines multiple indicators, in screening beneficiaries of immunotherapy improved significantly.

However, the correlation mechanism between these peripheral blood inflammatory complex indicators and tumor prognosis is complex and needs to be further explored via basic experiments and clinical trials. Recent studies reported that chronic inflammation may trigger gastrointestinal cancer, which may be one of the reasons for the correlation between peripheral blood inflammation indicators and tumor prognosis [38]. Apart from the direct immune response to kill tumor cells, these biomarkers are also related to tumor immunostimulatory signals and the activation of effector cells.

Neutrophils are traditionally defined as short-lived myeloid cells with unique cracks. As a type of white blood cells, they exist in the form of nuclear and rank the top in terms of importance and quantity in blood circulation. They are usually the first responders under autoimmune physiological or pathological conditions of sterile injury, infection, and inflammation and act as the first line of defense to protect the host from tissue injury and infection [39,40]. Tumor-associated neutrophils (TAN) accumulate in local areas and can be triggered by external stimuli in the tumor microenvironment (TME), switching between an antitumorigenic phenotype and a pro-tumorigenic phenotype [41]. Neutrophils that promote tumor cell growth and metastasis have the following functions: direct cytotoxicity, secretion of reactive oxygen species (ROS), nitric oxide (NO) and proteases, regulation of reticulocytosis, autophagy and other immune cells [42]. These neutrophils can activate CD8+ T cells and DCS and may even present tumor antigens [43]. Antitumor neutrophils kill tumor cells through direct cytotoxic effects as well as indirect effects via the activation of adaptive immune responses [44]. Moreover, increased numbers of neutrophils can inhibit the immune effect ability of lymphocytes [45]. Lymphocytes are important cells in the body’s immune response, particularly responsible for adaptive immunity, providing antigen-specific responses regulated by class I major histocompatibility complex (MHC) [45,46]. Studies have shown that lymphocytes and their subsets (CD8+T cells and CD3+ T cells) are related to the good prognosis of some tumors [47,48,49]. Lymphocytes infiltrated in TME are usually an indicator of the body’s immune state, which can prevent the proliferation and migration of tumor cells [50].

The occurrence of tumor-related inflammation can inhibit the synthesis of albumin [51]. Meanwhile, with the decrease in serum albumin, tumor patients will suffer from decreased immunity and malnutrition [51,52]. Due to the lack of sufficient serum albumin combined with drugs, if patients with cancer develop hypoproteinemia, chemotherapy drugs may have high residues in the blood and high toxicity [52].

Studies have reported that low albumin concentration reflects cancer-induced malnutrition and may have a negative impact on prognosis [53]. In recent years, more and more researchers have paid attention to the combination of inflammatory cells representing systemic inflammatory state and albumin reflecting nutritional status.

Glasgow prognostic score (GPS), a composite biomarker of albumin and c-reactive protein (CRP), is reported to be a sensitive prognostic marker for GC [54]. Zhang J et al. found that a composite biomarker of serum CEA and fibrinogen/albumin ratio could also be used as a positive indicator to predict tumor progression and prognosis for GC patients [55]. As mentioned above, higher neutrophil levels and lower albumin levels likely cause a poor prognosis for cancer patients.

PNI, a composite index formed by ALC and albumin, has been proven to be related to the prognosis of advanced head and neck cancer treated with ICI [56]. PNI was initially established to evaluate the relationship between the baseline nutritional status of tumor patients undergoing gastrointestinal surgery and postoperative complications [57]. However, the correlation between the prognosis of PNI and ICI treatment is unclear. Ul M et al. retrospectively analyzed 99 patients with advanced stage IV head and neck tumors who received ICI treatment at Johns Hopkins Hospital from 2014 to 2020 and found that PNI and BMI were related to the prognosis of ICI treatment [57]. According to the optimal cut-off PNI value of 45, patients were divided into a low PNI (<45) group and a normal PNI (≥45) group. Compared with normal PNI, lower PNI significantly correlated with shorter OS (*p* = 0.014) and PFS (*p* = 0.016). After multivariate adjustment, PNI was still an independent prognostic factor of OS (*p* = 0.041) and PFS (*p* = 0.011) [57]. A retrospective study by Shoji et al. on 102 consecutive patients with NSCLC treated with ICI showed that the baseline PNI level was significantly correlated with PFS (*p* = 0.0013) and OS (*p* = 0.0053) [28]. The PFS and OS of the low-PNI group (<45.5) were significantly shorter than those of the high-PNI group (≥45.5) [28]. In addition, Peng et al. also proved that PNI is related to the prognosis of NSCLC patients receiving ICI treatment [58].

In our study, the average PNI of 43.44 is taken as the best cut-off value, which is close to the best cut-off value taken by Uller M et al., Shoji et al., and Peng et al. [28,57,58]. According to the optimal cut-off value determined using PNI, patients were divided into a high-level group and a low-level group. Our study shows that the prognosis of PNI treated with ICI in AGC patients is basically consistent with Uller M et al., Shoji et al., and Peng et al. The PFS and OS of the high-level group were longer than those of the low-level PNI group. Due to our included subjects using different immunotherapy regimens in different therapeutic lines, we further conducted subgroup analysis to explore the influence of PNI on OS and PFS in different lines and different immunotherapy regimens. Subgroup analysis showed that patients with high PNI could benefit from OS whether they used immunotherapy of the first line or the multi-line, but PFS could only benefit from immunotherapy of the first line. Moreover, patients in the high-PNI group can benefit from OS and PFS, both in immunotherapy alone and in combination with other regimens. Patients with a reduced PNI from baseline after 4 weeks of immunotherapy are at increased risk of recurrent tumor progression within 3 months, and those with a reduced PNI from baseline after 8 weeks of immunotherapy are at increased risk of death within 8 months. Our research also found that AGC patients with pleural fluid and subsequent ICIs cannot benefit from PFS and OS after immunotherapy. In addition, we observed an interesting phenomenon that the level of PNI significantly correlates with the patient’s age, ECOG PS, and smoking history. Younger patient age and lower ECOG PS lead to higher levels of PNI. We speculate that the reason for this result is that the younger and more physically fit the patient, the higher the level of albumin in the body and, therefore, the higher the resulting PNI level. But as to why patients with a history of smoking or even longer smoking duration would have higher PNI levels, we cannot explain exactly why. It is necessary to expand the sample size to verify the real link between the two.

## 5. Limitations

There are some limitations in this study, including but not limited to (1) the accuracy of the results may be undermined by retrospective bias such as selection, recall, and measurement; (2) patients included in the criteria received different drugs in different treatment lines; (3) the study subjects were confined to the same hospital; (4) the collected peripheral blood results could not reflect the actual dynamic changes; and (5) the exploration scope of this study was limited to peripheral blood, which was widely used in clinical practice and easy to operate, and did not involve genomics and radiomics, which might provide more valuable information and enrich the contents.

## 6. Conclusions

This study demonstrated that a composite biomarker of PNI was independently associated with the survival of AGC patients receiving immunotherapy. Compared to patients with malignant tumors with strong immunity, those with GC benefit less from immunotherapy. Although PD-1/PD-L1 expression level, microsatellite instability level, tumor mutation load, EBV, and other indicators significantly affect the efficacy of immunotherapy, these indicators cannot fulfill the criteria for accurate screening. Whether the peripheral blood composite index of PNI can be used as an effective and economical prognostic biomarker needs to be further investigated in future studies.

Immunotherapy is a new option for the treatment of GC. In future scientific studies, effective immunotherapy markers will be explored in multiple aspects combined with the results of genetic testing or immunohistochemistry to achieve an accurate treatment of GC. It is expected that more patients with AGC will benefit from the new ICIS-based treatment strategy.

## Figures and Tables

**Table 1:** General data and clinical features.

Characteristics	Number of Patients	Characteristics	Number of Patients
	Overall (n = 268)		Overall (n = 268)
Median age (range), years	59 (18–86)	Sex	
		Female	69 (25.7)
		Male	199 (74.3)
Smoking history		Smoking exposure	
Yes	101 (37.7)	<30 packs per year	48 (17.9)
No	167 (62.3)	≥30 packs per year	220 (82.1)
ECOG performance status		Family history	
≥2	16 (6)	Yes	101 (37.7)
0–1	252 (94)	No	167 (62.3)
Pleural fluid		Ascites	
Present	19 (7.1)	Present	66 (24.6)
Absent	249 (92.9)	Absent	202 (75.4)
Liver metastasis		Lines of immunotherapy	
Present	119 (44.4)	≥2	143 (53.4)
Absent	149 (55.6)	<2	125 (46.6)
Tumor_location		PD-1 inhibitor Combined with other therapies	
Cardia	73 (27.2)	Monotherapy	52 (19.4)
Body/Fundus	110 (41)	Chemotherapy	43 (16.0)
Pylorus	83 (31)	Target therapy	43 (16.0)
Unknown	2 (0.7)	Chemotherapy and target therapy	130 (48.5)
HER-2		PNI, mean (range)	
Present	34 (12.7)	≥43.44	149 (55.6)
Absent	177 (66)	<43.44	119 (44.4)
Unknown	57 (21.3)		

PD: progressive disease; HER-2: human epidermal growth factor receptor-2; PD-1: programmed cell death-1; ECOG PS: Eastern Cooperative Oncology Group performance status scores; ICIs: immune checkpoint inhibitors; PNI: the prognostic nutritional index.

**Table 2:** Relationship between PNI groups and response to anti-PD-1 treatment.

Best Overall Respons		Number of Patients (%)		*p* Value
	Overall n = 268	The Low-PNI Group n = 137	The High-PNI Group n = 131	
CR	4 (1.5)	0 (0)	4 (3.1)	0.236
PR	78 (29.1)	40 (29.2)	38 (29)	0.146
SD	75 (28)	33 (24.1)	42 (32.1)	0.973
PD	111 (41.4)	64 (46.7)	47 (35.9)	0.072
Objective response	82 (30.6)	40 (29.2)	42 (32.1)	0.611
Disease control rate	157 (58.6)	73 (53.3)	84 (64.1)	0.072

PNI: the prognostic nutritional index; PD-1: programmed cell death-1; CR: complete response; PR: partial response; SD: stable disease; PD: progressive disease.

**Table 3:** Logistic regression of disease control rate.

Patient Characteristics	Univariate Analysis OR (95%CI)	*p*	Multivariate Analysis OR (95%CI)	*p*
ECOG PS				
0–1	1 Reference		1 Reference	
≥2	0.299 (0.101–0.887)	0.029	0.462 (0.149–1.434)	0.181
Lines of immunotherapy				
<2	1 Reference		1 Reference	
≥2	0.367 (0.221–0.609)	<0.001	0.387 (0.231–0.648)	<0.001
Ascites				
Absent	1 Reference			
Present	0.803 (0.459–1.407)	0.444		
Pleural fluid				
Absent	1 Reference			
Present	0.771 (0.303–1.964)	0.586		
PNI, cut off (rang)				
≥43.44	1 Reference		1 Reference	
<43.44	0.581 (0.355–0.949)	0.030	0.640 (0.381–1.075)	0.092

**Table 4:** Association of δ PNI changes after 4 weeks (4 weeks—baseline) and recurrence after 3 months of immunotherapy.

Time	OR (95% CI) N = 220	*p* Value
4 weeks	0.954 (0.911–0.999)	0.043

**Table 5:** Association of δ PNI changes after 8 weeks (8 weeks—baseline) and death within 8 months of immunotherapy.

Time	OR (95% CI) N = 205	*p* Value
8 weeks	0.943 (0.898–0.989)	0.017

## Data Availability

The data presented in this study are available on request from the corresponding author. The data are not publicly available due to future planned analysis.

## Abbreviations

Gastric cancer (GC); median overall survival (mOS); advanced GC (AGC); overall survival (OS); chimeric antigen receptor T cells (CAR T cells); immune checkpoint inhibitors (ICIs); carcinoembryonic antigen (CEA); carbohydrate antigen (CA19-9); gastric cancer antigen (CA724); programmed death ligand-1 (PD-L1); microsatellite steady-state (MSI); lung immune prognostic index (LIPI); neutrophil-lymphocyte ratio (NLR); the prognostic nutritional index (PNI); platelet-lymphocyte ratio (PLR); hemoglobin (Hb); non-small cell lung cancer (NSCLC); eastern cooperative oncology group performance status scores (ECOG PS); complete response (CR); partial response (PR); stable disease (SD); progressive disease (PD); disease control rate (DCR); overall response rate (ORR); progression free survival (PFS); receiver operating characteristic (ROC); programmed death-1 (PD-1); Tumor-associated neutrophils (TAN); tumor microenvironment (TME); reactive oxygen species (ROS); nitric oxide (NO); major histocompatibility complex (MHC); glasgow prognostic score (GPS); c-reactive protein (CRP).
